# Forgetting the new locations of one’s keys: spatial-memory interference in Korsakoff’s amnesia

**DOI:** 10.1007/s00221-018-5266-7

**Published:** 2018-04-21

**Authors:** Albert Postma, Sascha G. Morel, Margot E. Slot, Erik Oudman, Roy P. C. Kessels

**Affiliations:** 1 Experimental Psychology, Helmholtz Institute, Utrecht University, Heidelberglaan 1, 3584 CS Utrecht, The Netherlands; 2 Korsakoff Center Slingedael, Rotterdam, The Netherlands; 3 Centre of Excellence for Korsakoff and Alcohol-Related Cognitive Disorders, Vincent van Gogh Institute for Psychiatry, Venray, The Netherlands; 4 Donders Institute for Brain, Cognition and Behaviour, Radboud University, Nijmegen, The Netherlands; 5 Department of Medical Psychology, Radboud University Medical Center, Nijmegen, The Netherlands

**Keywords:** Object–location memory, Interference, Amnesia, Korsakoff’s syndrome

## Abstract

The present study focused on interference in a group of patients with amnesia due to Korsakoff’s syndrome (KS) within the domain of spatial memory. An object–location memory task was used in which participants first learned an array of objects on a computer screen, followed by a reconstruction of the object positions. Next a trial was given in which the same objects were presented only now in different locations. Participants had to place the objects a second time but at the new locations. This was repeated for seven pairs of baseline/interference trials. Both Korsakoff patients and matched controls did worse on the interference trials than on the baseline trials, indicating that it is difficult to relearn new spatial locations for objects that previously were remembered in other locations. When computing relative interference effects (that is the percentage change from baseline in the interference trials), Korsakoff patients were less affected than controls. It is discussed in how far interference depends on the strength of the original memories, which are markedly lower in KS patients.

## Introduction

Interference has long been identified as one the prime reasons for memory failure (McGeoch 1932; see Oberauer and Lin 2017, for its role in visual working memory). Competition between items in memory may cause sincere problems in learning, storage and/ or retrieval. The possibility that amnestic patients suffer in particular from interference effects has received considerable attention (Cowan et al. 2004; Dewar et al. 2010, 2012; Humphreys 2001; Kopelman 2002; Shimamura et al. 1995; Mayes et al. 1994), although the results are mixed (see also Craig et al. 2016).

In the present study, we further investigated interference in a group of patients with amnesia due to Korsakoff’s syndrome (KS) within the domain of spatial memory. KS is a chronic disorder, characterized by severe cognitive dysfunction, caused by lesions in the diencephalon, notably the thalamus and mammillary bodies, due to chronic alcohol abuse and thiamine deficiency (Arts et al. 2017; Fama et al. 2012). Most pronounced are the memory deficits, including both anterograde and retrograde amnesia (Kopelman 1995). The amnesia involves specifically explicit memory whereas implicit memory appears relatively spared (Oudman et al. 2011, 2015; Postma et al. 2008b).

Although the amnesia in KS is severe, it is particularly relevant for rehabilitation purposes to know to what extent KS patients are still able to acquire new information, either explicitly or implicitly. For this reason we investigated in which manner interference affects learning of new spatial information in KS patients in the present study. Two types of interference are important here (cf. Radvansky 2011). One is labeled negative transfer. That is, prior knowledge can have a negative effect on the learning of new information, in particular when there is an overlap (e.g., items coming from the same categories or sharing physical resemblances). Notice that negative transfer in particular plays a role in skill learning where it may yield its effects mostly in an implicit manner (Woltz et al. 2000). Second, there is proactive interference: the forgetting of new information because of preexisting older memories. Whereas negative transfer causes interference by hampering the acquisition phase, proactive interference influences the later processing stages of storage or retrieval.

Several studies have examined the factors that hamper memory for new information in KS patients. Cermak et al. (1977) observed that filling a delay with interfering activities impaired memory of KS patients for both verbal and nonverbal material. Strauss and Butler (1978) showed that KS patients performed worse than an alcoholic control group in a haptic (i.e., active touch) memory task when various types of interference were presented. However, these two studies are examples of distraction or dual tasks effects, rather than of proactive interference proper or of negative transfer. Kinsbourne and Winocur (1980) found evidence for stronger negative transfer in KS patients on a list-learning task containing word pairs. If the first half of the word pairs were coupled with new words when learning a second list, the patients’ performance significantly dropped. The authors explained their findings in terms of encoding difficulties in the new learning situation. Winocur et al. (1981) reported a similar buildup of proactive interference effects in KS patients when testing recall of successive lists of nouns from the same category. Release of proactive interference was achieved by introducing a category shift, but KS patients still needed additional cues in order for release of interference to occur compared to controls.

An important question is whether interference works differently for spatial memory. In his elegant paper on interference in spatial memory, Elmes (1988) discusses first the theoretical notion following from the work by O’Keefe and Nadel (1978) that the allocentric cognitive mapping system is insusceptible to interference by similar items. That is, we actually may profit from exploring the same locations, landmarks and roads from different directions when building an allocentric environmental map. In contrast, the taxon system (important for egocentric route representations) might strongly suffer from interference. In four experiments, Elmes (1988) observed considerable inference effects for learning and relearning of spatial mazes and for the locations of pairs of cards in a ‘concentration’ game.

Object–location memory differs from navigation in that there is no specific route or sequential aspect involved. Moreover, the spatial range typically is smaller (Postma and van der Ham 2016). Interference effects may be specifically large in object–location memory. This concerns the question how easily we can remember the new location of an object that first occupied a different location. Either proactive interference or negative transfer could hamper performance in these situations that are highly common in our daily life. That is, our personal belongings are typically small, moveable objects that can easily shift from one location to another in our house or living space. The inability to effectively store these new object locations may result in long, tiresome searches. In a classical neuropsychological study, Smith et al. (1995) showed series of pictures of objects in spatial arrays to patients with lesions in the medial-temporal lobe, including the hippocampus, patients with lesions in the prefrontal cortex and control participants. After the study phase, the participants had to place the pictures at the correct locations in an empty array. Next, the same objects were shown again, but in a new spatial arrangement. Larger proactive interference effects were found in both the prefrontal and medial-temporal lobe patients compared to controls.

In KS patients, who have diencephalic dysfunction, deficits in object–location memory have been well-documented (Kessels and Kopelman 2012; Kessels et al. 2000; Mayes et al. 1991; Postma et al. 2006, 2008b; Shoqeirat and Mayes 1991), but proactive interference or negative transfer has been examined to a lesser degree in this domain and in these patients. In this light, the goal of the present study was to further investigate the patterns of interference in an object–location memory paradigm in amnesic patients with KS. We analyzed both positional memory and object–location binding scores (cf. Postma et al. 2008a). We also computed relative difference scores between baseline and interference trials in order to assess the extent of interference (cf. Smith et al. 1995). Finally, we assessed whether interference-based errors were biased towards the spatial coordinates of the inducing (baseline) stimulus.

## Methods

### Participants

Twenty patients diagnosed with KS participated in this study (12 men). They were all inpatients of the Centre of Excellence for Korsakoff and Alcohol-Related Cognitive Disorders of Vincent van Gogh Institute for Psychiatry in Venray, the Netherlands. For all patients, the current intelligence level of each participant had to be in concordance with the estimation of premorbid functioning based on occupational and educational history to exclude cases of dementia (Oslin et al. 1998). Two patients (1 male) with estimated IQ scores below 80 were excluded because of low intellectual functioning interfering with the testing procedure, possibly caused by alcohol-related dementia. The remaining 18 patients (11 men) and 19 (6 men) age-, IQ- and education-matched controls were included in the analysis. All patients fulfilled the DSM-5 criteria for alcohol-induced major neurocognitive disorder (American Psychiatric Association 2013), supported by neurological, psychiatric, neuropsychological examinations, as well as neuroimaging findings. All patients also fulfilled the clinical criteria for KS described by Kopelman (2002). All patients were in the chronic, amnestic stage of the syndrome, whereas none of the patients was in the confusional Wernicke psychosis at the moment of testing. Moreover, every patient had been abstinent from alcohol for at least 6 weeks. Patients had an extensive history of alcoholism and nutritional depletion, notably thiamine deficiency, as verified through medical charts or family reports.

All patients completed the Dutch version of the California Verbal Learning Test (a task measuring verbal immediate and long-term memory; Mulder et al. 1996) and the Rivermead Behavioural Memory Test (RBMT; Wilson et al. 1985) as measures of episodic memory and the Corsi Block-Tapping Task (Kessels et al. 2000) as a measure of working memory.

Nineteen healthy control participants were included as well (6 men). None had a history of psychiatric disorders, neurologic disease or subjective cognitive complaints (self-report). For all participants education level was assessed using seven categories, one being the lowest (less than primary school) and seven being the highest (academic degree) (Duits and Kessels 2014). Premorbid IQ was estimated with the Dutch version of the National Adult Reading Test (Schmand et al. 1991). The study was approved by the Institutional Review Board of Vincent van Gogh Institute for Psychiatry and written informed consents were obtained at recruitment in all participants in accordance with the Declaration of Helsinki.

Table 1 summarizes the demographic variables of all participants and the neuropsychological test results and radiological findings for the patients. Controls and patients did not differ in age (*t*(35) = 0.04, *p* = 0.968), sex distribution (*χ*^2^(1) = 0.217, *p* = 0.642), education level (*t*(35) = 0.493, *p* = 0.625), or NART IQ (*t*(35) = 1.857, *p* = 0.072).

**Table 1:** Demographic variables, neuropsychological test results, and radiological finding of the Korsakoff’s patients

	Korsakoff patients (*N* = 18)	Healthy controls (*N* = 19)
Sex distribution (*m:f*)	11:7	6:13
Age (mean + SD)	50.7 (7.4)	50.8 (7.4)
Education level (mean + SD)^a^	4.5 (0.9)	4.6 (0.8)
NART IQ (mean + SD)^b^	96.5 (9.3)	101.8 (1.8)
California Verbal Learning Test^c^		
Severely impaired	12 (67%)	
Moderately impaired	2 (11%)	
Mildly impaired	4 (22%)	
Rivermead Behavioural Memory Test^d^		
Severely impaired	14 (78%)	
Moderately impaired	2 (11%)	
Mildly impaired	1 (5%)	
Corsi Block-Tapping Task (mean + SD)^e^	5.3 (0.93)	
Neuroimaging		
MRI	13	
CT	3	
Not available	2	
Neuroradiological findings		
Abnormalities in mammillary bodies	8	
Diffuse cortical atrophy	7	
Diffuse white matter lesions	4	
Cerebellar atrophy	3	
No abnormalities	3	

^a^Education level was scored using seven categories: 1 = lowest (less than primary school), 7 = highest (university degree) (Duits and Kessels 2014)

^b^NART = Dutch version of the National Adult Reading Test (Schmand et al. 1991)

^c^Based on the total score of the five learning trials, normative data from Mulder et al. (1996)

^d^Based on normative data from Van Balen et al. (1996). Note that the RBMT was not administered in one patient due to logistic reasons

^e^All patients performed within the normal range based on normative data from Kessels et al. (2000)

### Materials and procedure

**Fig. 1:**
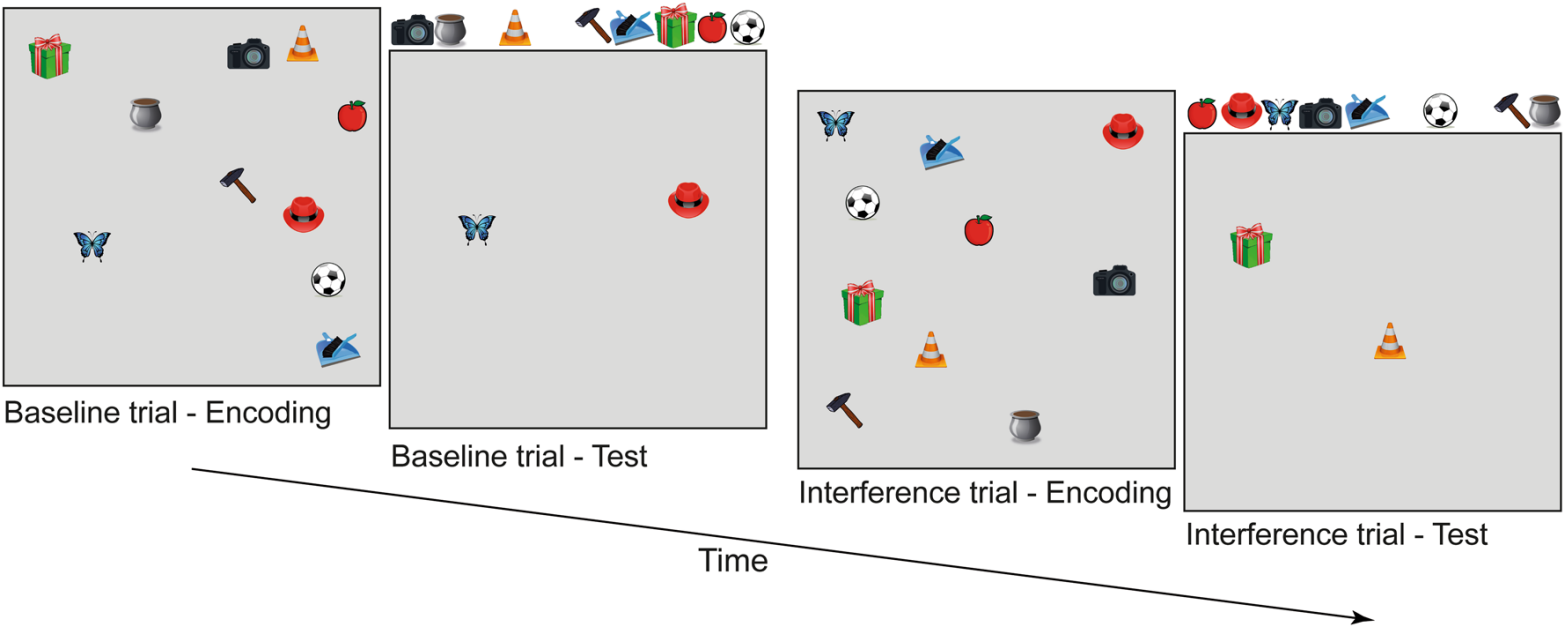
Schematic overview of two consecutive trials in the object–location memory task; participants are shown a display with common objects placed at pseudorandom locations, followed by a recall test in which objects have to be repositioned at their correct locations. Next, an interference trial is presented, showing the same objects, but at different locations. In the recall test, these objects have to be placed at the locations they occupied in the last presentation

Object–location memory was assessed by means of the computer program Object Relocation (Kessels et al. 1999). In each trial, ten colored images of easy-to-name everyday objects—measuring approximately 1 × 1 cm—were presented for 30 s on a computer screen within a square frame measuring 19 × 19 cm. Next, an empty square frame was shown with the 10 objects displayed on a row above the frame. Participants were instructed to reposition the objects at their initial locations. Once an object was placed in the frame it could be moved to other locations as often as a participant desired. The object–location memory stimuli were shown on a 15-in. Elo Entuitive monitor with touch-sensitive screen. Figure 1 gives a schematic overview of two consecutive trials (baseline and interference trial).

The object–location memory test started with a practice trial in which only four objects were shown. Next, 7 blocks of 2 object–location memory trials with 10 objects each followed. The first trial of a block always was a baseline trial, containing a completely new set of objects placed at new positions. The second trial of a block was the interference trial. Here, the same objects were shown as in the preceding baseline trial, but at new positions. Spatial configurations of baseline and interference trials were made by an automatic generation procedure of the object–location memory program (open to minor manual adjustments such as placing objects not too closely together). All participants received the same trials in a fixed order.^1^

### Analyses

To determine the accuracy of the locations of the repositioned objects, the absolute displacement error was computed. This is the absolute distance between the original object location and the reconstructed object location in mm, summed over the 10 objects in the stimulus display. A second spatial error measure was computed as well: the positional best-fit score, reflecting how accurately the positional configurations were reconstructed, irrespective of the object identities (Kessels et al. 1999). The absolute errors scores and best-fit scores were analyzed separately in 2 × 2 repeated-measures analyses of variance (ANOVA), with Group as a between-subjects factor (two levels: KS patients vs. healthy controls) and Task condition (two levels: baseline vs. interference) as within-subject factor. Effect sizes were computed for all analyses (*η*_p_^2^).

We also computed a percentage difference score, both for the absolute error and the best-fit scores, loosely based on the procedure reported by Smith et al. (1995, p. 281) as a measure of relative interference. This percentage difference score is the difference between the raw score (i.e., either the absolute displacement error or the best-fit score) on an interference trial and the performance on the corresponding baseline trial, expressed as the percentage change with respect to the baseline performance. For each participant relative interference scores were thus computed, averaged across all trial blocks. Independent *t* tests were conducted on the relative interference scores for the absolute error performance and for the best-fit performance, including group (KS patients, controls) as between-subject factor. Pearson product moment correlation coefficients (*r*) were computed between baseline errors and the relative interference scores.

Finally, we computed a third type of performance measure. We calculated a new absolute displacement error by using the original object location coordinates from the baseline trial and the coordinates of the corresponding objects in the interference trial, as reconstructed by the participant. Moreover, we also calculated a similar new absolute displacement error by taking the original object locations from the interference trial and the reconstructed positions of the corresponding objects in the baseline trial. Since the spatial configurations for the baseline and interference trials were made in (quasi)random fashion the combination of *interference trial original locations* and *baseline reconstructed locations* can be considered to reflect chance performance in the object–location memory task. In contrast, the combination *baseline original locations* and *interference trial reconstructed locations* might possibly show some lingering influence of the baseline spatial configuration on the subsequently repositioned objects in the interference trial. This may be a measure of an implicit memory influence across baseline and interference within trials. An implicit spatial-memory effect is reflected by lower new absolute displacement errors in the combination *baseline original locations*–*interference trial reconstructed locations* than in the chance performance combination (i.e., combination *interference trial original locations–baseline reconstructed locations*).

## Results

A 2 × 2 ANOVA, including condition (baseline, interference) as a within subjects factor and group (KS patients, controls) as a between-subjects factor, was conducted on the absolute error scores. It revealed a main condition effect (*F*[1, 35] = 37.7, *p* < 0.001, *η*_p_^2^ = 0.52), indicating that errors on the interference trials were higher than on the baseline trials. Moreover, the main effect of group (*F*[1, 35] = 36.2, *p* < 0.001, *η*_p_^2^ = 0.51) confirmed that KS patients overall performed worse compared to healthy controls. Notably, the condition × group interaction was not statistically significant.

**Fig. 2:**
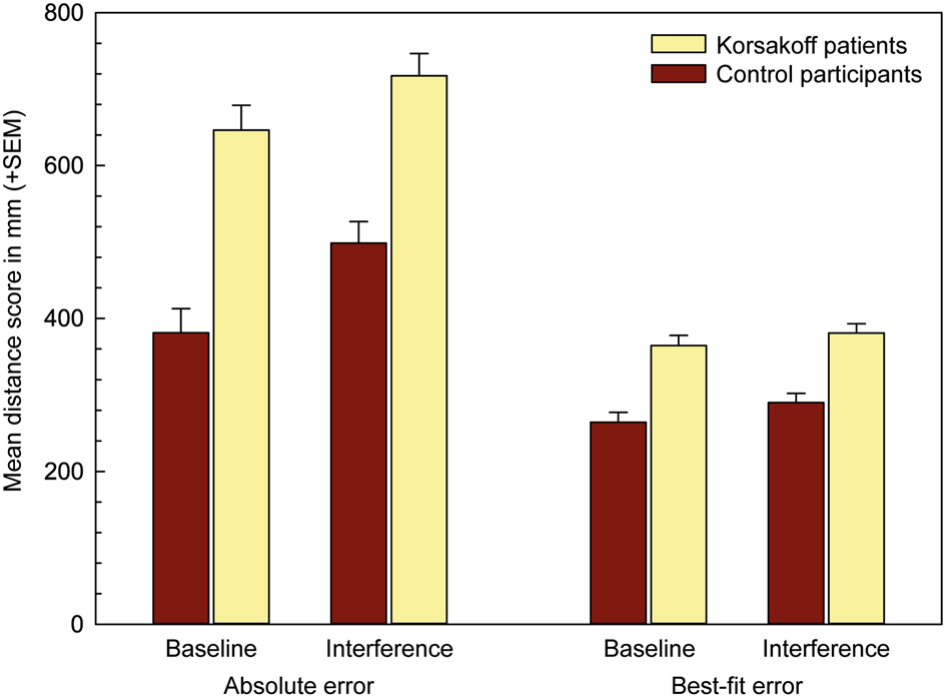
Spatial-memory scores in mm (summed over 10 objects in a stimulus display, averaged across stimuli) across condition (baseline and interference) and per group (controls vs. Korsakoff patients). Error bars indicate standard errors

Running a similar ANOVA on the best-fit scores showed also a main effect of condition (*F*[1, 35] = 9.99, *p* < 0.01, *η*_p_^2^ = 0.22) and a main effect of group (*F*[1, 35] = 32.92, *p* < 0.001, *η*_p_^2^ = 0.49). Best-fit scores were worse in the interference than in the baseline trials and worse in the KS patients than in the controls. Again, the condition × group interaction was not statistically significant (Fig. 2).

**Fig. 3:**
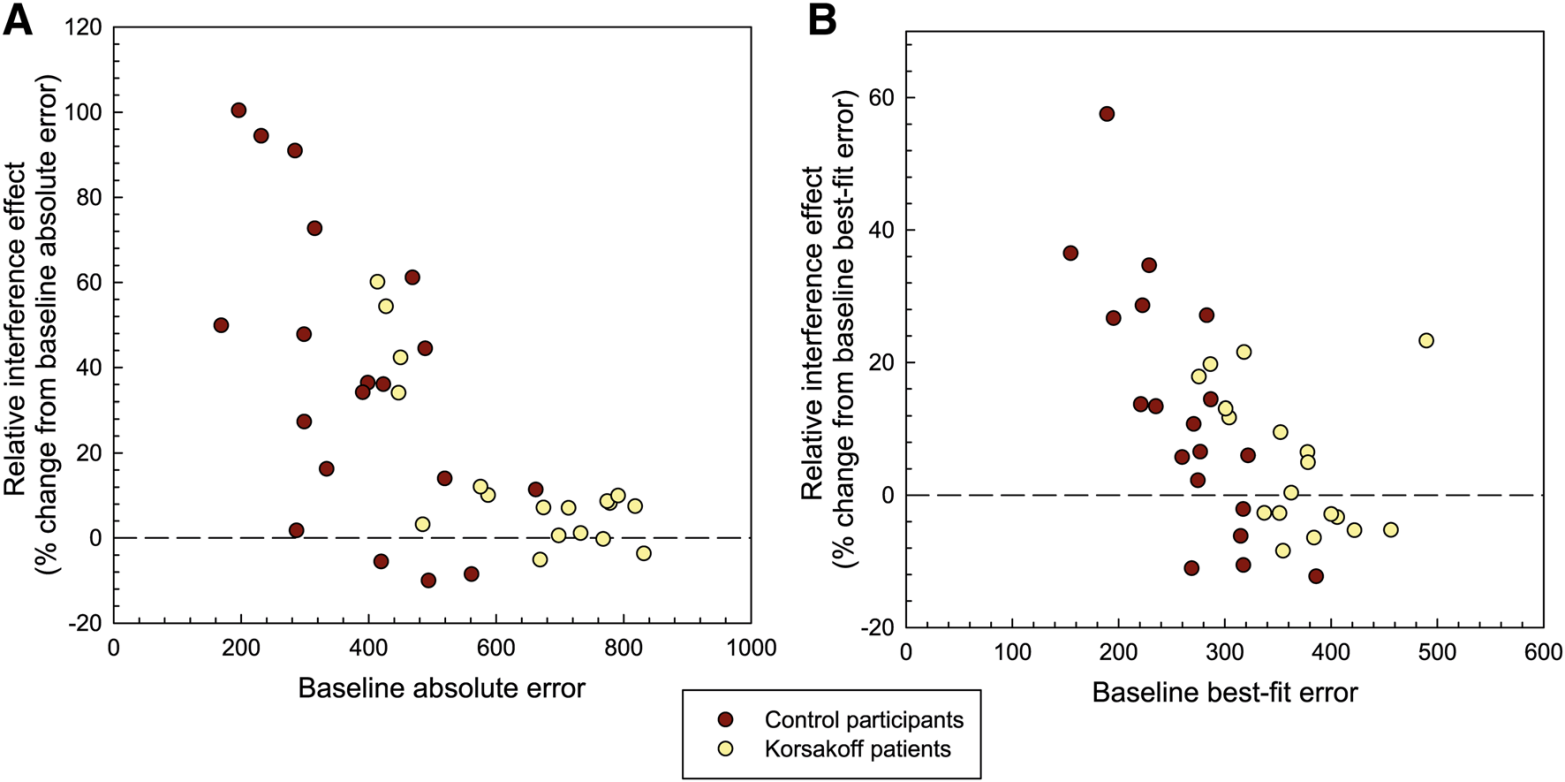
Scatter plots showing the relative interference effect as a function of baseline performance (i.e., distance errors in mm; sum of 10 objects in a stimulus display, averaged across stimuli) for the absolute error scores (**a**) and the best-fit error scores (**b**)

Figure 3 shows the scatterplots of the relative interference scores for the absolute and best-fit error scores, respectively. For both the absolute errors and the best-fit score, a negative correlation was found between baseline performance and relative interference effect, suggesting that stronger memory at baseline results in relatively more interference (*r*[37] = − 0.69, *p* < 0.01 for the absolute errors; *r*[37] = − 0.61, *p* < 0.01, for the best-fit scores). When computing these correlations for the separate groups, similar correlations were observed in the control group (*r*[19] = − 0.61, *p* < 0.01 for the absolute errors; *r*[19] = − 0.79, *p* < 0.01, for the best-fit scores). In the patient group baseline performance and relative interference effect correlated significantly (*r*[18] = − 0.78, *p* < 0.01) for the absolute errors, but not for the best-fit scores (*r*[18] = − 0.35, ns).

In order to compare the groups on relative interference levels, we conducted two separate independent *t* tests. There was a group effect for the relative interference effects on the absolute errors *t*[35] = 2.51, *p* < 0.05 (14.3% in KS patients; 37.6% in controls). Relative interference was more than twice as high in controls than in KS patients. No such difference was observed for the best-fit scores *t*[35] = 1.52, ns (5.1% in KS patients vs. 12.7% in controls, respectively). One could argue that the maximum relative increase from baseline that can be reached is limited by how close the baseline performance is to chance performance. In other words, if a participant already has a poor baseline score from the start, there may not be enough room to become much worse in the interference condition. As we argue below, chance level is 916 mm. A number of the KS patients indeed has average baseline absolute errors of around 800 mm (see Fig. 3a). Importantly, several of them do not show any interference-based error increase at all or only a very limited one. Even though the maximum relative interference increase is technically limited in KS patients, there still is room for a larger increase than the 14% increase we observed now (if patients would have had an increase of 37%, similar to that of the controls, their average interference scores would have been 889 mm).^2^

Finally, we compared the two groups on the reordered spatial distance scores, that is reconstructed locations in the baseline trials linked to the original place coordinates in the interference trials (KS patients mean 914.8 mm, SEM 8.9; control mean 918.3 mm, SEM 8.7), and vice versa reconstructed locations in the interference trials coupled to the original baseline coordinates (KS patients mean 843.6 mm, SEM 17.7; control mean 833.4 mm, SEM 17.3). We found that the former combination yielded higher scores than the latter (*F*[1, 35] = 43.18, *p* < 0.001, *η*_p_^2^ = 0.55). However, neither the group effect nor the group by recombined scores interaction was statistically significant. As we argued above, the combination of original interference trial coordinates with baseline reposition coordinates might give an estimate of chance performance on this type of task. The average chance level over all participants was: 916 mm, SE 6.1, minimum 861 mm, maximum 993 mm. Both the small standard error and the limited range of values suggest that the average value of 916 might be an appropriate approximation of chance. We next contrasted KS patients’ absolute errors performance on the baseline trials and the interference trials with chance. Both performances differed significantly from chance (*t*[17] = 8.1, *p* < 0.001 for the baseline trials and *t*[17] = 7.8, *p* < 0.001 for the interference trials, respectively).

## Discussion

Memory for spatial context information is compromised in KS patients (Kessels et al. 2000; Postma et al. 2006, 2008b; Shoqeirat and Mayes 1991). The current study examined whether heightened susceptibility to interference (i.e., how well patients can associate new locations with ‘old’ objects) underlies these spatial-memory deficits. More specifically, we investigated mechanisms of proactive interference or negative transfer in object–location memory in KS patients.

A first prerequisite in this investigation was that we observed interference effects within the present experimental design. Indeed we found that the overall performance dropped substantially when having to learn new locations for objects that were previously studied and tested in combination with other locations. In the introduction we argued that this reflects either proactive or negative transfer. We cannot really differentiate between the two options here. In light of the fact that the different trials directly followed each other with only limited time in between, we are inclined to think that mostly the encoding of new location information is at stake here. Hence the interference most likely reflects negative transfer.

Central in the current study was the comparison between KS patients and matched controls with respect to interference effects in object–location memory. Two patterns of results bear on this comparison. First, the raw spatial-memory scores showed that both controls and KS patients suffered from interference effects, but did not significantly differ in the size of these effects. Second, in contrast to the findings for the raw spatial-memory scores we did observe a clear group difference when looking at the *relative size* of the interference effect (the percentage change compared to baseline), with controls being relatively more affected by the interference manipulation. It also appeared that the relative interference effect is directly driven by the strength of the first established memory trace, as indicated by the (negative) correlation between the two. We should mention here that these correlations are somewhat flawed, since the baseline performance level is also used to compute the relative interference score (hence it features twice in the correlation). Notwithstanding this reason for caution, the pattern seems to suggest that KS patients, having weaker memories from the start, may have a relative benefit when having to learn new object–location associations.

In light of this last suggestion, it is interesting to consider how control participants deal with forming new associations. Possibly, they need to actively suppress the old associations during the interference trials. This mechanism might be similar to the active inhibitory process revealed in the motivated forgetting paradigm (Anderson and Hanslmayr 2014), recruiting the right dorsolateral prefrontal cortex. In other words, to counter interference by strong existing memories dorsolateral prefrontal control processes are required. KS patients are known to have impaired control processing or executive function deficits. Consequently, we expect that under circumstances requiring executive inhibitory control (that is, situations with strong baseline memories) they will show enlarged interference effects.

Interestingly, the group difference with respect to the relative interference effect exclusively applied to the absolute error scores, but not to the best-fit scores. KS patients demonstrated a smaller relative interference effect than the controls. The absolute errors reflect a process of binding objects to locations (e.g., remembering ‘what was where’), whereas best-fit scores reflect positional memory per se (e.g., remembering which positions in a display were occupied) (Postma et al. 2008a). Apparently it is the formation of new object–location associations that drives the interference effects and the group differences observed in the present study. Future studies may investigate whether interference also occurs in the reverse situation: old locations occupied by new objects across trials. If not, it would signal that object–location associations and location–object associations are asymmetric, distinct processes.

Are KS patients affected on all dimensions of object–location memory? In the present study, we also computed the errors scores when coupling repositioned coordinates on the interference trials to the corresponding object coordinates on the baseline trials and compared this to the reverse coupling (i.e., baseline repositions with interference trials’ original object coordinates). We observed smaller error scores in the former recombination. This marks a subtle influence of the first object–location pattern learned on the next trial. We speculate that this influence is most likely implicit, as participants are typically deliberately and consciously focusing on the new locations in the current (interference) trial. Importantly KS patients did not differ from controls on these implicit spatial-memory scores. This is in agreement with previous evidence for spared implicit spatial memory in KS patients (Oudman et al. 2011, 2015; Postma et al. 2008a, b). Together with the lower relative interference scores in patients, the interference effects in the present paradigm thus may be driven by the competition between old and new memory traces at the explicit level.

In sum, the present study confirmed a large deficit in KS patients in associating objects with locations. This deficit is not due to increased sensitivity to proactive and/or negative transfer interference effects. The negative transfer to some extent depends on the strength of the old memories, which are markedly lower in KS patients. As a result, learning of new information is relatively less affected. A critical future test will be to see whether strengthening the original memory traces (e.g., by repeated or longer learning; see also Shoqeirat and Mayes 1991) may increase relative interference effects in KS patients at or past the level encountered in healthy controls.^3^

## Footnotes

1 By giving a fixed stimulus order, we ensured that the difficulty of the task was exactly the same for the two groups. As pointed out by an anonymous reviewer, there may have been some a priori differences between the spatial layouts used in the baseline stimuli and those used in the interference stimuli. However, the stimulus displays were created in (quasi)random fashion. We examined whether the study-phase spatial layouts of the baseline stimuli differed from those in the interference trials, taking the object locations as independent units (70 in the baseline stimuli and 70 in the interference stimuli). There were no differences between the two stimulus types with respect to the spatial coordinates used in the study phase, making systematic a priori differences between the baseline and interference trials unlikely.

2 We thank an anonymous reviewer for bringing up this point. Of course other technical error limitations are related to the size of the square frame, since participants cannot place objects outside the square. Hence the maximum distance error occurs when an object is placed in the opposite furthest corner of the square frame. For all the locations chosen in the baseline stimuli this distance was 1970 mm (i.e., summed over the 10 locations in a stimulus) and 1980 mm for the locations in the interference stimuli.

3 We are grateful to Prof. Michael D. Kopelman for bringing this suggestion to our attention during a conference presentation.
